# *Toxoplasma gondii* GRA15 DNA Vaccine with a Liposomal Nanocarrier Composed of an SS-Cleavable and pH-Activated Lipid-like Material Induces Protective Immunity against Toxoplasmosis in Mice

**DOI:** 10.3390/vaccines10010021

**Published:** 2021-12-24

**Authors:** Tanjila Hasan, Ryo Kawanishi, Hidetaka Akita, Yoshifumi Nishikawa

**Affiliations:** 1 National Research Center for Protozoan Diseases, Obihiro University of Agriculture and Veterinary Medicine, Inada-cho, Obihiro 080-8555, Hokkaido, Japan; tanjila@cvasu.ac.bd (T.H.); madeinobihiro@yahoo.co.jp (R.K.); 2 Department of Medicine and Surgery, Faculty of Veterinary Medicine, Chattogram Veterinary and Animal Sciences University, Khulshi, Chattogram 4225, Bangladesh; 3 Laboratory of DDS Design and Drug Disposition, Graduate School of Pharmaceutical Sciences, Chiba University, Chiba City 260-0856, Chiba, Japan; akitahide@chiba-u.jp

**Keywords:** DNA vaccine, lipid nanoparticle, TgGRA15

## Abstract

*Toxoplasma gondii* affects the health of humans and livestock and causes severe illness in the fetus and immunocompromised individuals. Because of the high incidence and severe consequences of *T. gondii* infection, a safe and suitable vaccine is needed. We found that lipid nanoparticles (LNPs) consisting of a series of functional materials prepared with vitamin E, such as SS-cleavable and pH-activated lipid-like materials (ssPalmE), were a safe and efficient way to develop next-generation DNA vaccines. In this study, we prepared ssPalmE-LNP to encapsulate pCpG-free-*T. gondii* dense granule protein 15 DNA (ssPalmE-LNP_TgGRA15_). Following a challenge infection with avirulent PLK strain of *T. gondii*, the mice immunized with ssPalmE-LNP_TgGRA15_ had a significantly higher survival rate and lower clinical scores compared with unimmunized and ssPalmE-LNP_non-coding_-immunized mice. Immunization of mice with the ssPalmE-LNP_TgGRA15_ led to a significantly higher production of specific IgG1 and IG2c antibodies compared with unimmunized and ssPalmE-LNP_non-coding_-immunized mice, while there was no statistically significant difference in the concentration of serum interferon-gamma at the acute stage of the infection. These findings indicate that ssPalmE-LNP is an effective cargo for the transportation of DNA vaccines for protozoan infections. To explore the mechanism of protective immunity induced by ssPalmE-LNP_TgGRA15_, further immunological study is needed in the future.

## 1. Introduction

Toxoplasmosis is caused by *Toxoplasma gondii*, an apicomplexan, opportunistic protozoan parasite, and is a global public health concern [1]. In immunocompetent individuals, the disease is mostly asymptomatic or subclinical, but is fatal in immunodeficient individuals because it can affect the liver, brain, eyes, and other organs [2,3]. In pregnant women, it can cause miscarriage, perinatal death, or fetal organ abnormalities, depending on the trimester of pregnancy [4]. Toxoplasmosis is responsible for devastating economic losses in the livestock industry because it can induce abortion and stillbirth in livestock, and promote cyst formation in meat [5,6], which impacts national and international trade markets [7]. However, despite these economic and zoonotic concerns, there are currently no effective vaccines or treatments available for toxoplasmosis. Therefore, an effective vaccine that can induce strong immune protection against *T. gondii* infection in humans and animals is needed [8].

In recent years, vaccines produced for *T. gondii* have included inactivated or attenuated vaccines, protein vaccines, and DNA vaccines, but their overall protective efficacies have been unsatisfactory [9]. DNA vaccines are simple to produce, inexpensive, highly effective, heat stable, convenient, and safe [10]. Moreover, these types of vaccines have the ability to elicit cellular and humoral immune responses [11]. Furthermore, DNA vaccines are considered an important potential tool for the defense against *T. gondii* because they can induce CD8^+^ T cell-mediated cellular immunity [12]. However, DNA vaccines are easily degraded by extracellular nucleases [13], undergo minimal cellular uptake, and are known to undergo inadequate endosomal/lysosomal escape [14]. To overcome this problem, lipid nanoparticles (LNPs) that consist of several functional components, including an SS-cleavable and pH-activated lipid-like substance (ssPalm), have been developed as vaccine carriers. The advantage of using LNPs is that once the LNP–vaccine complex enters a cell, the LNPs are spontaneously degraded and the vaccine cargo is released into the intracellular environment [15]. Kawai et al. [16] used ssPalmE-LNP as an adjuvant for a protein vaccine and observed strong cytotoxic T lymphocyte (CTL) activity against cancer. Moreover, Maeta et al. [17] found that mice immunized with plasmid DNA (pDNA) encoding ovalbumin, encapsulated by ssPalmE-LNPs, as well as a *T. gondii* profilin antigen, exhibited significant antitumor and antiprotozoan responses. 

*T. gondii* dense granule protein 15 (TgGRA15) is a novel effector protein that is essential for nuclear factor-kappa-B (NF-κB) translocation and transcriptional activity. TgGRA15 can modulate the NF-κB pathway, which is dependent on TNF receptor-associated factor 6 (TRAF6) and the inhibitor of nuclear factor-κB kinase (IKK) complex, resulting in the production of inflammatory cytokines, such as interleukin-12 (IL-12) and IL-1β [18,19]. In Type I, Type II, and Type III *T. gondii* infection, Type II TgGRA15 was found to have higher potential for host NF-κB signaling pathway activation compared with Type I and Type III TgGRA15s [18]. Together, TgGRA15 and ssPalmE-LNP can activate the immune system. Therefore, in our present study, we generated TgGRA15-encoded ssPalmE-LNP (ssPalmE-LNP_TgGRA15_) as a DNA vaccine to determine its efficacy to provide immune protection in a mouse model. 

## 2. Materials and Methods

### 2.1. Ethics Statement

All the surgical procedures including infection to mice, in this experiment, were performed under general anesthesia using isoflurane to reduce the pain and stress of the mice. We strictly obeyed the rules and regulations and guidelines that are provided by the Care and Use of Laboratory Animals of the Ministry of Education, Culture, Sports, Science and Technology, Japan. Afterward, the experimental design was approved by the Committee on the Ethics of Animal Experiments at the Obihiro University of Agriculture and Veterinary Medicine (permission numbers 27–30).

### 2.2. Mice

Eight-week-old, female C57BL/6J mice were purchased from CLEA Japan (Tokyo, Japan). The mice were kept in a pathogen-free environment in accordance with the National Research Center for Protozoan Diseases at Obihiro University of Agriculture and Veterinary Medicine in Obihiro, Japan. 

### 2.3. Cultures and Purification of T. gondii

The PLK strain of Type II *T. gondii* was maintained in monkey kidney-adherent fibroblasts (Vero cells) and cultured in Eagle’s minimum essential medium (Sigma, St. Louis, MO, USA) supplemented with 8% heat-inactivated fetal bovine serum (Nichirei Biosciences, Tokyo, Japan) and antibiotics (1% streptomycin-penicillin, Sigma, St. Louis, MO, USA). For purification of tachyzoites, the infected cells were syringe-lysed using a 27-gauge needle to release the tachyzoite-stage parasites into the medium, which was then filtered using a 5.0 μm pore-sized filter (Millipore, Bedford, MA, USA). 

### 2.4. Cloning of the TgGRA15 Gene

To generate a construct that expressed the FLAG-tag-fused TgGRA15 from Type II *T. gondii*, TgGRA15 cDNA was amplified by PCR from *T. gondii* cDNA using the forward primer 5′-ACC AGT CGA CTC TAG ATG GTG ACA ACA ACC ACG CC-3′ and the reverse primer 5′-AGT CAG CCC GGG ATC TTG GAG TTA CCG CTG ATT GT-3′, and cloned into *Xba*I and *Bam*HI sites of p3×FLAG-CMV-14 (Sigma) by In-Fusion cloning (Takara Bio Inc., Shiga, Japan) to generate p3XFLAG-CMV-TgGRA15. Finally, the open reading frame of TgGRA15 was inserted into the *Bgl*II and *Xba*I restriction enzyme sites of pCpG-free-NEWmcs [20] to prepare pCpG-free-TgGRA15. Regarding the non-coding pDNA, pCpG-free-MCS (Invivogen, San Diego, CA, USA) was used.

### 2.5. Preparation of LNP-Encapsulated pCpG-free-TgGRA15

pCpG-free-TgGRA15 encapsulation with LNPs was prepared by the ethanol dilution method, as defined by Maeta et al. [17], leading to the generation of ssPalmE-LNP_TgGRA15_. Briefly, 1,2-Dioleoyl-sn-glycero-3-phosphoethanolamine (DOPE) was purchased from Avanti Polar Lipids (Alabaster, AL, USA). Cholesterol was purchased from Sigma Aldrich (St. Louis, MO, USA). The 1-(Monomethoxy poly (ethylene glycol)2000)-2,3-dimyristoylglycerol (DMG-PEG2000) and ssPalm with vitamin E as a hydrophobic scaffold (ssPalmE: COATSOME^®^ SS-E) were purchased from NOF corporation (Tokyo, Japan). Protamine sulfate salmon milt was purchased from Calbiochem (Darmstadt, Germany). The LNP encapsulating DNA were prepared by formation of pDNA/protamine complex, followed by encapsulation in the LNP (ssPalmE/DOPE/Chol/DMG-PEG2000 = 3/4/3/0.3). The size of the ssPalmE-LNP particle was 140 nm, and slightly negatively charged (approximately −5 mV). Moreover, the encapsulation efficiency was >90%.

### 2.6. Immunization and Infection in Mice

Mice were immunized with 10 µg ssPalmE-LNP_non-coding_ (*n* = 10) or 10 µg ssPalmE-LNP_TgGRA15_ (*n* = 10) via subcutaneous injection (100 μL, administration volume). Additionally, 100 μL of ultrapure water was injected subcutaneously as an immunization vehicle in a separate group of mice (Milli-Q; *n* = 8). On day 0, day 14, and day 28, all mice from each group were immunized (a total of three immunizations per mouse). The sera from all mice were collected via the tail vein 3 days before the first immunization and 7 days after each immunization. The sera were kept at −20 °C until use. All mice from each group were challenged with 3 × 10^3^ PLK strain via intraperitoneal injection 2 weeks after the third immunization. Survival observations of the mice were recorded for 30 days post-infection (dpi).

### 2.7. Measurement of TgGRA15-Specific Antibodies

The levels of TgGRA15-specific immunoglobulin G (IgG), IgG1, and IgG2c were measured in mouse sera with an enzyme-linked immunosorbent assay (ELISA). To prepare the antigens for ELISA, 293T cells were transiently transfected with the p3XFLAG-CMV-TgGRA15 [21] with FuGENE HD transfection reagent (Promega, Madison, WI, USA), according to the manufacturer’s instructions. Non-transfected cells were used as a control. At 20 h post transfection, cells were harvested and treated with lysis buffer (50 mM Tris-HCl pH 8.0, 125 mM NaCl, 0.25% NP-40) for 1 h on ice. After centrifugation at 10,000× *g* for 10 min at 4 °C, the supernatants were collected. Protein concentrations in the supernatant were measured using the bicinchoninic acid protein assay kit (Thermo Fisher Scientific Inc., Rockford, IL, USA). Thereafter, 1 μg of protein from the cell extracts was mixed with 50 μL carbonate–bicarbonate buffer (pH 9.6) and coated onto the ELISA plates (Nunc, Denmark) overnight at 4 °C. The ELISA was performed as described previously by Terkawi et al. [22]. The plates were incubated with 50 μL aliquots of sera from the immunized or control mice (diluted 1:200), which were added to the wells in duplicate, followed by incubation with horseradish peroxidase-conjugated goat anti-mouse IgG, IgG1, or IgG2c (diluted 1:10,000).

### 2.8. Measurement of IFN-Gamma (IFN-γ)

The serum was collected from individual mouse at 7 dpi to measure IFN-γ by ELISA kits (Mouse OptEIA ELISA set; BD Biosciences, San Jose, CA, USA) according to the manufacturer’s instructions. The optical density was measured using an ELISA reader at 450 nm.

### 2.9. Clinical Score

To determine the severity of infection, a modified SHIRPA protocol was followed to assess clinical scores [23]. One score was allocated for each clinical sign such as piloerection, hunching, huddling, ptosis, sunken eye, latency in movement, gait, no reflex/alert, flaccid, and loss of balance. Each of the following changes received one point. A score ranging from 0 (no signs) to 10 (all signs) was assigned to the average reported clinical signs. The clinical scores were counted for each mouse from −2 (two days before infection) to 14 dpi [24]. 

### 2.10. Statistical Analysis

Survival curves were generated using the Kaplan–Meier method, and statistical comparisons were made using the log-rank method. Statistical significance for the comparison of antibody levels was analyzed using one-way analysis of variance (ANOVA) followed by Tukey’s multiple comparison test. GraphPad Prism version 5 (GraphPad Software Inc., La Jolla, CA, USA) was used for all statistical analyses. Statistical significance was described as a *p*-value less than 0.05.

## 3. Results

**Figure 1:**
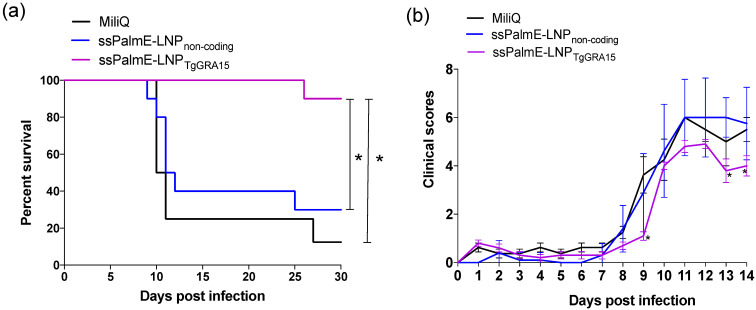
Survival rates and clinical scores of immunized mice after challenge with *T. gondii*. The mice were immunized with ssPalmE-LNP_TgGRA15_ (*n* = 10), ssPalmE-LNP_non-coding_ (*n* = 10), or Milli-Q water (*n* = 8), and then challenged with the PLK strain of *T. gondii*. (**a**) Survival curves were generated using the Kaplan–Meier method. *, The significance of differences in mouse survival was analyzed by the log-rank test. The differences were significant between Milli-Q water-injected and ssPalmE-LNP_TgGRA15_-immunized groups, and ssPalmE-LNP_non-coding_-injected and ssPalmE-LNP_TgGRA15_-immunized groups (* *p* < 0.05). (**b**) Clinical scores were monitored daily up until 14 days after *T. gondii* infection. The mean ± SD represents the average clinical score values of all mice in each group. Statistical significance was determined by two-way ANOVA and Bonferroni post hoc analysis (* p < 0.05) for ssPalmE-LNP_TgGRA15_-immunized groups compared with the ssPalmE-LNP_non-coding_-injected and Milli-Q water-injected groups.

In the challenge infection, mice immunized with ssPalmE-LNP_TgGRA15_ had a significantly higher survival rate (9/10; 90%) compared with Milli-Q water-injected mice (1/8; 12.5%) and ssPalmE-LNP_non-coding_-injected mice (3/10; 30%; Figure 1a). Moreover, mice immunized with ssPalmE-LNP_TgGRA15_ had significantly lower clinical scores at 9, 13, and 14 dpi compared with the other groups (Figure 1b). 

**Figure 2:**
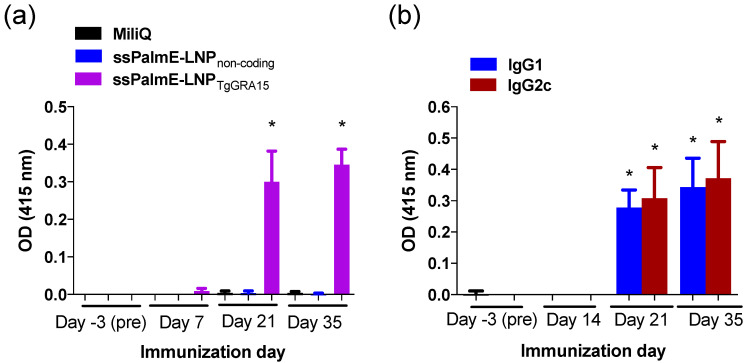
TgGRA15-specific antibody responses of immunized mice. (**a**) Sera from mice immunized with ssPalmE-LNP_TgGRA15_ (*n* = 10), ssPalmE-LNP_non-coding_ (*n* = 10), or Milli-Q water (*n* = 8) were analyzed using ELISAs to detect TgGRA15-specific total IgG in the sera. (**b**) Analysis of TgGRA15-specific IgG1 and IgG2c antibodies in the sera from mice immunized with ssPalmE-LNP_TgGRA15_ (*n* = 10). The values are expressed as optical densities at 415 nm. Each bar represents the mean ± SD. (*) significant differences of the same day (**a**) and same antibody subclass (**b**), which were determined by one-way ANOVA and a Tukey post hoc test (* *p* < 0.05).

Mice immunized with ssPalmE-LNP_TgGRA15_ developed high levels of anti-TgGRA15 IgG antibody after the second immunization (Figure 2a). Additionally, both IgG1 and IgG2c antibodies against TgGRA15 were detected after the second immunization (Figure 2b). 

**Figure 3:**
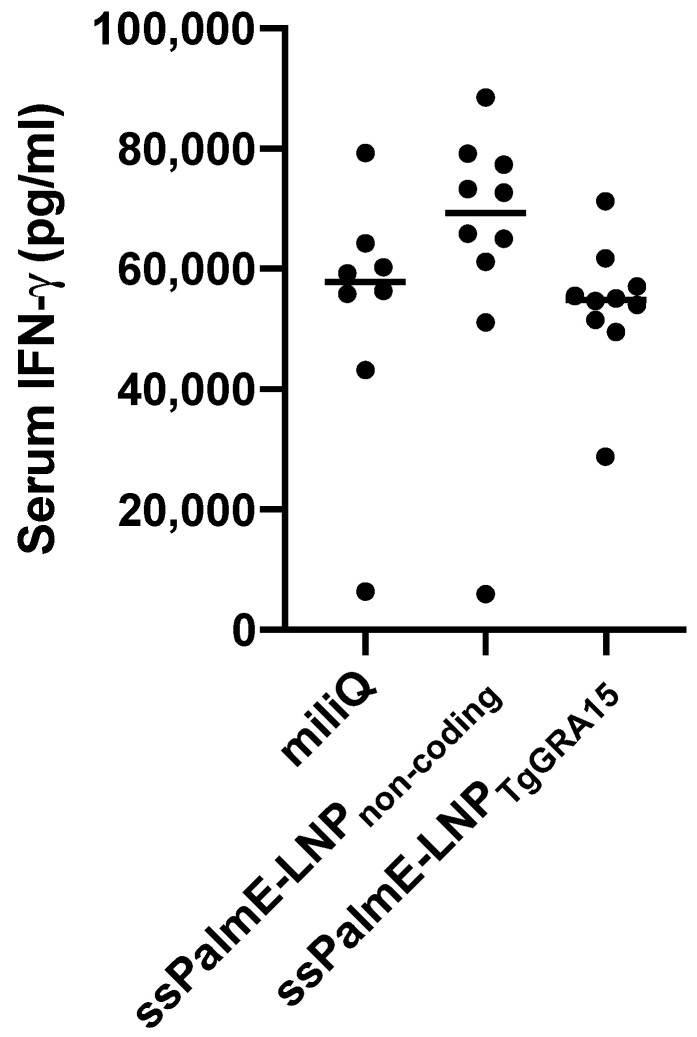
Measurement of IFN-γ in serum of immunized mice at 7 days post infection. Sera from mice immunized with ssPalmE-LNP_TgGRA15_ (*n* = 10), ssPalmE-LNP_non-coding_ (*n* = 10), or Milli-Q water (*n* = 8) were analyzed using ELISA. The values are expressed as optical densities at 450 nm. The results were analyzing using one-way ANOVA and a Tukey–Kramer post hoc analysis, but there was no significant difference.

To evaluate the cellular immune response, we measured serum IFN-γ at 7 days after the challenge infection (Figure 3). However, there was no significant difference in IFN-γ concentration among the experimental groups.

## 4. Discussion

**Figure 4:**
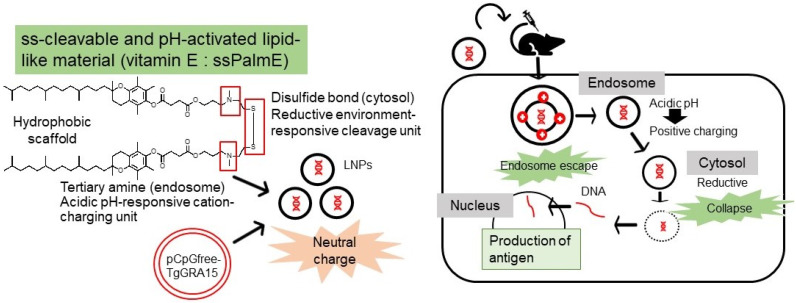
Mechanism of action of ssPalmE-LNP_TgGRA15._ ssPalm contains two hydrophobic scaffolds to form a stable lipid bilayer. For membrane destabilization, proton-sponge units are positively charged at an acidic pH (endosome/lysosome) in the two tertiary amines. In reducing environment (cytosol), disulfide bonding is cleaved. Following cellular uptake and in response to the intracellular environment, the LNP is degraded, which leads to the release of the cargo or antigen (TgGRA15).

In recent years, there has been considerable investigation into potential approaches to improve the efficacy of DNA vaccines, for instance by reducing extracellular nuclease degradation or by removing the endosomal or lysosomal membrane (Figure 4). Vaccines developed with LNP-encapsulated pDNA may be an effective approach to overcome the above-mentioned problems [15]. ssPalm also contains two hydrophobic scaffolds to form a stable lipid bilayer. For membrane destabilization, proton-sponge units are positively charged at an acidic pH (endosome/lysosome) in the two tertiary amines. In addition, in a reducing environment (cytosol), disulfide bonding can be cleaved. Following cellular uptake and in response to the intracellular environment, the LNP is degraded, which leads to the release of the cargo or antigen [15]. ssPalms with different hydrophobic scaffolds, such as myristic acid (ssPalmM), retinoic acid (ssPalmA) [25], and α-tocopherol (ssPalmE) [26], have been used. Of these, ssPalmE was found to be the most effective for the delivery of different nucleic acids, such as short interfering RNA (siRNA) and pDNA, to the liver when administered intravenously [16]. Some studies suggest that the use of vitamin E as a hydrophobic scaffold may increase the antigen concentrations in monocytes, lead to a higher accumulation of granulocytes in lymph nodes, and promote the production of Th1-type cytokines [27]. A study by Kawai et al. [16] indicated that ssPalmE-LNP had a greater interferon-β-producing ability in RAW 264.7 cells compared with ssPalmM-LNP and could evoke antigen-specific cytotoxic T cell activity when injected subcutaneously. Moreover, Maeta et al. [17] found that administration of ovalbumin with ssPalmE-LNP_non-coding pDNA_ strongly activated CTL activity compared with other ssPalm-LNPs. 

Compared with other GRAs, GRA15 has recently received significant attention because of its potent role in the host–pathogen interaction. GRA15 has been detected at the parasitophorous vacuole, as well as in the host cell cytoplasm [18]. TgGRA15 activates NF-κB signaling pathways, likely via activation of TRAF6, which in turn activates IKK, leading to the phosphorylation and degradation of inhibitor of NF-κB. This facilitates the subsequent translocation of NF-κB to the nucleus to induce the expression of a broad range of genes involved in the immune response and inflammation [28]. GRA15 also activates the STING pathway via TRAF proteins, which in turn enhances defensive mechanisms against *T. gondii* infection [29]. This immune response is responsible for reducing parasitic tissue invasion, increasing host survival, and assisting conversion of the parasite into a bradyzoite cyst [30]. Mice infected with a GRA15II-deficient strain were unable to produce significant levels of IFN-γ and showed severe defects in both NF-κB nuclear translocation and NF-κB-mediated transcription, and had increased susceptibility to infection [18,21]. 

Both host humoral and cellular immunity play an important role in the defensive mechanism against toxoplasmosis. It is notable that infection by *T. gondii* can typically induce a persistent and strong immune response, which is partially composed of high-level cytokine and IgG antibody levels [31]. Among different cytokines, IFN-γ exhibits a crucial role by stopping parasite growth in the acute stage. In our present study, serum IFN-γ was assayed in the immunized and the non-immunized mice. The concentration of IFN-γ was not different significantly among the experimental groups, although the survival rate of the vaccinated mice was significantly high. This might be due to the production of a significant amount of anti-*Toxoplasma*-specific IgG1 and IgG2c antibodies that induce a humoral immune response, resulting in the inhibition of the tachyzoite invasion into the target cells [32]. Furthermore, Chen et al. [33] reported that immunization of mice with pVAX-GRA15 resulted in significantly increased serum IgG2a titers. Moreover, the authors found a link between Th1 responses and the production of IFN-γ, IL-2, IL-12 p40, and IL-12 p70, implying that DNA vaccination has a significant protective efficacy. The estimation of IgG2c levels is one of the indicators for the Th1-type immune response because there is a correlation between IgG2c and IFN-γ production in C57BL/6 mice [34,35]. Liu et al. [36] stated that mice immunized with pROP16-GRA7 had higher IgG titers, increased IFN-γ secretion, and a higher percentage of CD8^+^ T cells. In addition, DNA vaccination against *T. gondii* SAG1 [37,38], as well as *T. gondii* GRA1, GRA7, and ROP2 [39], induced the production of specific IgG1 and IgG2a antibodies and protective immunity. According to Echeverria et al. [40], Rop2-83 immunization in C57BL/6 mice resulted in IgG1/IgG2c production and reduced cyst load. In our study, anti-GRA15 IgG1 and IgG2c antibodies were observed in mice vaccinated with ssPalmE-LNP_TgGRA15_, suggesting an induction of humoral and Th1-type immune responses at the site of infection.

Furthermore, the role of specific anti-TgGRA15 antibodies in TgGRA15-induced defense was verified by the fact that mice immunized with these antibodies survived longer than control mice [33]. In this study, TgGRA15 was encapsulated by ssPalmE-LNP, which acted as a vehicle. The ssPalmE-LNP was able to induce anti-*T. gondii* and antitumor immunity when injected subcutaneously as a DNA vaccine [17], which also supports the results of our study. 

To control toxoplasmosis and induce a protective immune response, the combination of humoral and cellular immune response is effective. However, in our present study, we mainly focused on the production of antibody production and it was found that the mice vaccinated with ssPalmE-LNP_TgGRA15_ could produce anti-GRA15 IgG1 and IgG2c antibodies, but no significant difference in serum IFN-γ concentration at the acute stage of the infection was seen. To explore the mechanism of protective immunity induced by ssPalmE-LNP_TgGRA15_, further immunological study is needed in the future.

## 5. Conclusions

The development of an effective vaccine against *T. gondii* remains a matter of concern. The results of our study demonstrate that immunization of mice with ssPalmE-LNP_TgGRA15_ as a DNA vaccine is sufficient to produce anti-*T. gondii*-specific IgGs, including IgG1 and IgG2c, which are indicative of protective efficacy (Figure 4). Further studies investigating the combination of TgGRA15 with other antigens to determine potential combinations that may elicit stronger immune protection in both pregnant and non-pregnant models are required.

## Data Availability

The research data can be acquired on request from the correspondence author (principal investigator of the project).
